# A targeted tiled amplicon sequencing approach for clade and subclade level differentiation of monkeypox virus from wastewater

**DOI:** 10.1038/s41598-025-13927-y

**Published:** 2025-08-11

**Authors:** Mathew Fisher, Jennifer Ali, Shayna Giesbrecht, Raveena Roopra, Veronica Carroll, Amber Papineau, Cody Buchanan, Nicholas Duan, Chrystal Landgraff

**Affiliations:** 1 Wastewater Genomics Unit, Bacterial Pathogens, AMR, and Wastewater, National Microbiology Laboratory, Public Health Agency of Canada, Winnipeg, MB Canada; 2 Department of Food Science, Ontario Agricultural College, University of Guelph, Guelph, ON Canada

**Keywords:** Tiled amplicon sequencing, MPOX, Wastewater, Monkeypox virus, Molecular biology, Infectious diseases, Viral infection, Comparative genomics

## Abstract

Wastewater-based surveillance (WBS) has modernized in recent years and emerged as an important tool for the monitoring of viral pathogens, including monkeypox virus (MPXV). Here we describe a novel targeted amplicon sequencing method developed for clade and subclade characterization of MPXV from municipal wastewater. This new method addresses the limitations of PCR-based methods and the challenges of sequencing a pathogen displaying low viral load in municipal wastewater samples. A tiled amplicon scheme composed of 11 primer pairs targeting a 4.2 kb portion of the inverted terminal repeat (ITR) region of the MPXV genome was designed and tested. In silico analysis demonstrated high accuracy for clade and subclade calls using the full target region, with specific amplicons also exhibiting strong performance individually. An MPXV consensus sequence representing the entire target region was successfully sequenced from a wastewater sample and differentiated from positive controls by a distinct deletion within a short homopolymeric region. Notably, clade-informing data was also achieved from partial sequences recovered from lower abundance samples. This study presents a new sequencing method targeting MPXV with enhanced genomic resolution compared to existing PCR-based approaches, providing critical genomic-level information informing MPXV surveillance and public health interventions.

## Introduction

Monkeypox virus (MPXV) is an enveloped, double-stranded DNA virus with an approximate genome size of 197 kb and the causative agent of a zoonotic disease called mpox (formerly known as monkeypox). Along with the closely related variola virus, the etiological agent responsible for smallpox, it belongs to the *Orthopoxvirus* genus. It was first described in 1957 following an outbreak in a Danish monkey colony^1^ with the first human cases detected in the Democratic Republic of the Congo (DRC) in 1970 during the smallpox eradication campaigns^2^. For the next 50 years it was considered a rare zoonotic disease, endemic to the Central and West African regions and human cases were typically attributed to contact with infected animals. Historically, MPXV has caused rare sporadic disease in African countries up until 2003 when it caused a notable outbreak in the United States due to direct contact with infected prairie dogs imported from Ghana^3^. This outbreak represented the first MPXV infections outside of the African continent until its re-emergence in 2022 resulting in a global epidemic.

From July 2022 until May 2023, the World Health Organization (WHO) declared a public health emergency of international concern (PHEIC)^4^ due to the rapid increase in mpox cases and spread of a new clade of MPXV clade Ib from countries outside of West Africa where clade II is endemic, to non-endemic countries including the United Kingdom^5^, Canada^6^ and the USA^7^. By May 2023, over 88,060 laboratory-confirmed cases and 147 deaths were reported across 120 countries^8^ with the most significantly impacted demographic of the epidemic being men who have sex with men. Although the PHEIC phase of this public health event was declared over in May 2023, clade IIb continued to transmit and cases were detected worldwide. In September 2023, a new outbreak event was detected, emerging from the South Kivu region of the DRC, eventually prompting the WHO declaration of a second PHEIC on August 14, 2024^9^. A main contributing factor leading to the declaration of this second PHEIC was the emergence of a new clade I strain designated as clade Ib^9^. The global public health community, including Canada’s disease surveillance programs are exercising increased vigilance concerning clade Ib, as clade I is considered more virulent than clade II. However, a recent WHO study found only a slight difference at 0.19% and 0.7%, respectively, in the mortality rates of clade IIb and clade Ib lineages responsible for the 2022-23 and 2023-ongoing outbreaks^10^. Recently, travel associated cases of clade Ib have been reported in a number of countries outside of Africa including Canada^11^, the United States^12^, Germany^13^, India^14^, Sweden^15^, Thailand^16^ and the United Kingdom^17^.

Wastewater-based surveillance (WBS) is an active surveillance strategy that was significantly advanced and deployed for community-level surveillance throughout the coronavirus disease 2019 (COVID-19) pandemic and during that time, demonstrated capacity as a leading indicator of disease incidence^18,19^. Many studies have demonstrated a complementarity role of WBS to traditional, clinical pathogen surveillance, and its advantageous^20–22^, functions as an unbiased and cost-effective disease monitoring approach that also captures signals shed by infected individuals, including from those displaying mild symptoms or asymptomatic cases. Furthermore, community-level studies have revealed a high correlation between the quantity of viral nucleic acid recovered from wastewater to reported clinical case numbers^23^. Beyond the COVID-19 pandemic, WBS has been expanded to understand the transmission dynamics of other viral pathogens circulating within a population including influenza virus^24–28^, respiratory syncytial virus^27–29^, MPXV^30–34^ and dengue virus^35^.

As communities across Canada and globally implement monitoring for the importation of MPXV, including clade Ib, PCR methods targeting a variety of genomic targets have been developed for wastewater testing. However, PCR methods are inherently limited in the amount of genomic information they produce, hindering their capacity for taxonomic classification or rapid detection of new or emerging clades, subclades, or genomic variants. As a result, genomic sequencing can bridge these surveillance gaps. Although several assays are now available for MPXV sequencing, these methods target the complete genome and were developed for and tested on purified clinical specimens. Wastewater presents a unique matrix and with it, unique challenges that may hamper the ease-of-adoption of assays designed for sequencing of clinical specimens. Unlike most clinical specimens, wastewater is a microbially mixed matrix potentially composed of multiple, diverse pathogens shed by a catchment population into an eventual wastewater treatment plant. The detection of a targeted pathogen by PCR or sequencing is further complicated if the pathogen is not abundantly shed, if it is not present in high copy numbers in the feces or urine of infected individuals or if there are low numbers of infected individuals within a wastewater treatment plant’s catchment area.

Here, we describe a novel targeted sequencing method and its successful use to detect and differentiate MPXV signals from municipal wastewater samples to the clade and subclade level. Although alternative whole genome targeted sequencing approaches were initially tested^36^, including a commercially-available assay, these assays had been developed for clinical sequencing and did not yield MPXV sequences from the wastewater samples tested, likely due to the lower viral load compared to clinical samples and the large amplicon size (> 5 kb). For this reason, we developed a novel set of tiled amplicon sequencing primers, targeting a portion of the genomically variable ITR region^37,38^ located at the terminal end of the MPXV genome. Similar tiled amplicon sequencing methods have been described in previous studies for a variety of targets including the widely used ARTIC scheme for SARS-CoV-2 sequencing^39,40^. The method presented in this study improves upon the data obtained by PCR-based methods by providing increased genomic resolution and enabling not only detection of MPXV but also clade and subclade level characterization.

## Materials and methods

### Wastewater sample collection

**Fig. 1:**
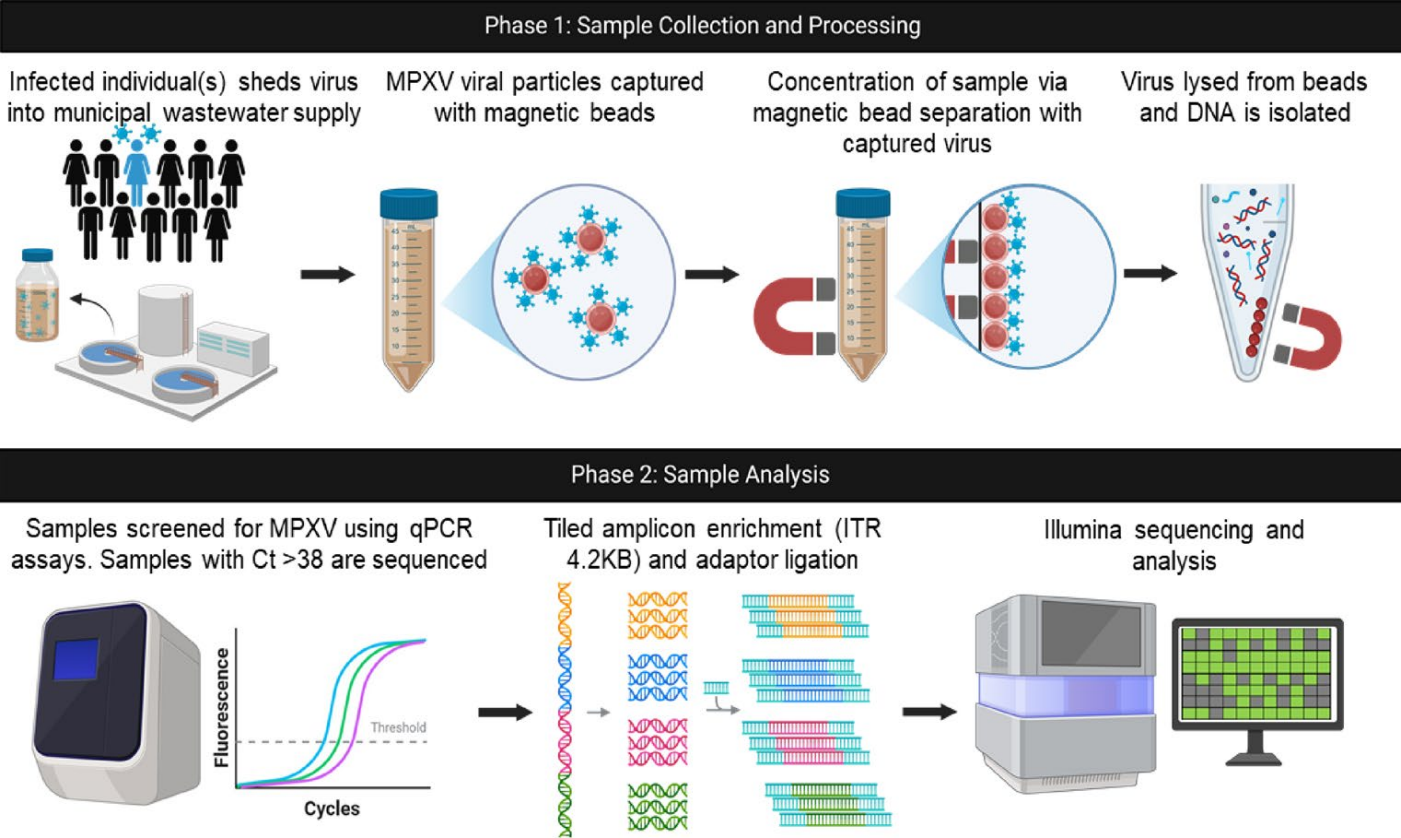
Overview of the wastewater collection, sample processing and analysis workflow used in this study.

Twenty-four hour composite influent wastewater from four different wastewater treatment plant sites in a large Canadian city with a combined catchment population nearing three million were used in this study (Fig. 1). Composite wastewater influent samples from each site were collected using an autosampler on a biweekly basis from September to October of 2024 as a total composite volume of 400–500 ml. Sterile polyethylene terephthalate bottles were used for collection and storage. Samples were shipped to the JC Wilt Infectious Diseases Research Centre in Winnipeg, Manitoba on ice and stored at 4 °C upon receipt until processing.

### Capture and concentration of MPXV

Initial attempts to concentrate MPXV from wastewater samples were performed using Nanotrap^®^ microbiome A particles (Ceres Nanosciences, Virginia, USA) according to manufacturer described methods using 40 mL of wastewater, a procedure currently in use within our laboratory for SARS-CoV-2. Due to a lack of recovery of MPXV nucleic acid, the starting volume of wastewater influent was increased to 120 mL which similarly did not capture sufficient material for a sequencing reaction. In response, a review of the published literature identified additional concentration methods for capture of MPXV from wastewater, including polyethylene glycol (PEG) precipitation of total suspended solids and extraction of the pellet fraction^41,42^. Several attempts to rapidly identify a suitable wastewater processing method were unsuccessful, prompting a shift from processing of the liquid fraction or suspended solids to capture of MPXV from total wastewater solids. This method, adapted from Mejia et al. (2024)^30^, consisted of centrifuging 120 mL of wastewater for 30 min at 12,000 x g to generate a more solid pellet. The supernatant was removed and the pellet was lysed by addition of 800 µL of CD buffer at room temperature followed by nucleic acid extraction using the Qiagen MagAttract PowerMicrobiome DNA/RNA KF extraction kit (Cat. No. 27600-4-KF) with a final elution into 100 µl of buffer EB. To improve the sequencing yield, a clean-up step was added using a 1:1 ratio of AMPure XP beads for DNA cleanup (Beckman Coulter, Brea, California, USA, Cat. No. A63881), 75% ethanol for washes, and 100 µl of Qiagen buffer EB for the final elution step. Detailed protocols of each of the above methods is shown in Figure S1. All results described in this study were obtained using the method designated as Method F (Figure S1) for wastewater processing and nucleic acid extraction prior to amplicon sequencing using the newly designed tiled primer schema presented in this study targeting a 4.2-kb genomic ITR region.

### Real time quantitative polymerase chain reaction (RT-qPCR)

To determine the presence of MPXV in wastewater, samples were screened prior to sequencing using previously published RT-qPCR assays (G2R_G^43^, G2R_NML^30^ and F3L^44^. PrimeTime Probes (Integrated DNA Technologies) were synthesized with a 5’ 6-FAM fluorophore and 3’ ZEN-Iowa Black FQ quencher. Primer and probe concentrations are as described in the literature^30,31,43^. Each PCR reaction was performed in triplicate in a total volume of 20 µL using the QuantiNova Multiplex PCR Kit (Qiagen, 208452) according to the manufacturer’s specifications. The QuantStudio 5 Real-Time PCR System (Applied Biosystems, A28138) was used with the following conditions: 95 °C for 2 min, followed by 42 cycles of amplification at 95 °C for 5 s and 60 °C for 30 s. Samples with a cycle threshold value (C_T_) of less than 38 for any one of the three assays were selected for sequencing. PCR assay positive controls consisted of both a high and low concentration of gBlocks^®^ Gene Fragments (Integrated DNA Technologies) for each PCR target. Positive controls included in all runs consisted of gBlock^®^ Gene Fragments (Integrated DNA Technologies) containing the fragment of interest (gBlock sequences and other information available in Table S1) at final concentrations of 10 cp/µL and 1000 cp/µL and diluted AMPLIRUN Monkeypox Virus DNA Control at a final concentration of 35 cp/ml (Vircell Molecular, MBC146-R).

### Quantification of DNA standards

The initial concentration of each gBlocks^®^ Gene Fragment (Integrated DNA Technologies) was quantified by digital PCR according to the manufacturer’s recommendations. Briefly, each gene fragment was diluted in nuclease-free water to a calculated concentration of 10,000 cp/ µL and tested in quadruplicate. A reaction mix volume of 9 µL was prepared using Absolute Q DNA Digital PCR MasterMix (5X) (Applied Biosystems, A52490), primer and probe concentrations of 100 µM, and 2 µL of each diluted gene fragment. Absolute quantification was performed using the QuantStudio Absolute Q Digital PCR System (Applied Biosystems, A52864) under the following conditions: activation for 10 min at 96 °C followed by 40 cycles of 96 °C for 5 s and 60 °C for 15 s.

### Generation of standard curves and determination of PCR assay sensitivity

A six-point standard curve from 5 to 500,000 copies per reaction was generated for each qPCR assay using quantified, serially diluted gBlock Gene Fragments (Integrated DNA Technologies) containing the target sequence of the G2R_G, G2R_NML and F3L RT-qPCR assays. Six replicates of each concentration were used per target. The sensitivity of each PCR assay was assessed using serially diluted gBlock Gene Fragments (Integrated DNA Technologies) containing 0.78 to 200 copies per reaction. Twenty replicates of each concentration were tested and a cycle threshold value of less than 40 was interpreted as a positive detection. To calculate the limit of quantification (LOQ), only concentrations in which all replicates were positive were used. The coefficient of variation (CV) was calculated for each concentration tested and fit to a linear regression model linking it to the template concentration^45^. The LOQ was defined as the concentration at which a CV equal to 35% was achieved as predicted by linear regression^45^. To determine the limit of detection (LOD), results were converted into binary values (i.e. positive and negative detection only) and a probit model was generated to predict the relationship between template concentration and positive detection. The LOD was defined as the lowest concentration at which a positivity rate of 95% was maintained as predicted by the probit model. All modeling was performed in RStudio using the tidyverse set of packages^46^.

### Tiled MPXV amplicon scheme design

Initial analysis was performed on a database of complete MPXV genomes downloaded from GISAID which were selected to represent the genetic diversity of the virus, including both recent outbreak and historical sequences. To reduce the redundancy and overall size of the dataset for processing, CD-HIT^47^ was used to cluster and remove sequences with more than 95% sequence identity, resulting in a database of 49 MPXV genome sequences. The sequences were aligned with MAFFT^48^ and the resulting alignment was manually investigated for the presence of variable regions suitable for design of a tiled amplicon scheme allowing for clade and subclade differentiation. This process resulted in the identification of an approximately 4.2 kb portion of the ITR region that was extracted from the alignment for further analysis. CD-HIT^47^ was used to remove redundant sequences with greater than 99% identity, resulting in a final database of nine sequences spanning the target region which was used for subsequent assay design. A tiled amplicon scheme was designed with PrimalScheme v.1.3.2^49^ using the final database of nine MPXV sequences. The resulting scheme consisted of 11 primer pairs generating PCR products ranging from 490 to 516 bp (Table 1). The primers were mapped to a whole genome database of representative MPXV sequences using Geneious Prime v2025.0.2^50^ to screen for mismatches that could impact primer binding efficiency. This database consisted of recent whole genome sequences representing MPXV subclades from all global regions with collection dates starting January 1, 2023, downloaded from GISAID^51^ on November 20, 2024 with the “complete” and “low coverage excl” filters enabled. The only exception was clade IIa which had no sequences in the GISAID repository within the specified collection dates, so two historical sequences from the USA and Liberia isolated in 1962 and 1970 were used for analysis (GISAID accession EPI_ISL_13056556 and EPI_ISL_13058405, respectively). The sequences included are available on GISAID at 10.55876/gis8.250624tz or under ID EPI_SET_250624tz. A table of dataset information provided by GISAID as well as a list of GISAID accession numbers for all sequences used at the time of analysis are available as supplementary materials.

**Table 1:** MPXV 4.2 kb ITR tiled amplicon sequencing primers and amplicons. For each primer, two primer binding location ranges (with position values relative to reference NC_063383.1) are shown since the MPXV genome contains two ITRs and therefore each primer has two separate binding locations.

Amplicon size (bp)	Primer name	Primer sequence (5’ to 3’)	Primer binding coordinates
516	hMpxV_J1-J3_1_LEFT	TAACGCATTTATGGACGACGGT	4,530–4,551; 192,659 − 192,680
	hMpxV_J1-J3_1_RIGHT	TGGAACGCGGATATGTGTTTACA	4,036 − 4,058; 193,152–193,174
495	hMpxV_J1-J3_2_LEFT	ACAAATTATTGACTAAAGGATCTGACCC	4,102–4,129; 193,081–193,108
	hMpxV_J1-J3_2_RIGHT	ACCGTAGTATATTGAGAGAGCGACT	3,635–3,659; 193,551 − 193,575
506	hMpxV_J1-J3_3_LEFT	TGGCACGAACAAAAATACGGGA	3,725–3,746; 193,464 − 193,485
	hMpxV_J1-J3_3_RIGHT	ACACTTTTATAGTCCTCGTTTAAACAGA	3,241–3,268; 193,942 − 193,969
510	hMpxV_J1-J3_4_LEFT	TCTCCCTACGACGATCACTACG	3,332–3,353; 193,857 − 193,878
	hMpxV_J1-J3_4_RIGHT	TGGATAATATTTGTAATGGTTCTTTCCGT	2,844–2,872; 194,338 − 194,366
492	hMpxV_J1-J3_5_LEFT	GACTATCGTATTTGCCTCCGGA	2,929–2,950; 194,260 − 194,281
	hMpxV_J1-J3_5_RIGHT	CGCGTCTCTACCTGATTACTATCAC	2,459–2,483; 194,727 − 194,751
509	hMpxV_J1-J3_6_LEFT	CGTGTGGTTCGGATACCTTTACA	2,530–2,552; 194,658 − 194,680
	hMpxV_J1-J3_6_RIGHT	TGCTACATTATTAAGGACAGAGAAGTATTC	2,044 − 2,073; 195,137–195,166
498	hMpxV_J1-J3_7_LEFT	AACTATATCGATGTGGAAATTAACCTGT	2,193–2,220; 194,990 − 195,017
	hMpxV_J1-J3_7_RIGHT	GGAATTAGTGATCAGTTTATGTATATCGCA	1,723–1,752; 195,458 − 195,487
512	hMpxV_J1-J3_8_LEFT	AGCGTCGACATCTACATACTATATAGT	1,840–1,866; 195,344 − 195,370
	hMpxV_J1-J3_8_RIGHT	TCGGATACCTCATCATCTTCGGT	1,355–1,377; 195,833 − 195,855
491	hMpxV_J1-J3_9_LEFT	CACAAAGCAAGACCAAACACCG	1,420–1,441; 195,769 − 195,790
	hMpxV_J1-J3_9_RIGHT	CCTCACACATGTCTCCGATACG	951–972; 196,238 − 196,259
512	hMpxV_J1-J3_10_LEFT	TAGATTGTCCAGCGTGTCACC	1,202–1,222; 195,988 − 196,008
	hMpxV_J1-J3_10_RIGHT	GTAGTTAAATATTTTTGTTTTGCAAACCGG	711–740; 196,470 − 196,499
490	hMpxV_J1-J3_11_LEFT	TCATCTGAAAATGGATGAGTTGGGT	796–820; 196,390 − 196,414
	hMpxV_J1-J3_11_RIGHT	GAGCAGTGTCCCCTACATGGAT	331–352; 196,858 − 196,879

### In Silico tiled sequencing primer scheme evaluation

The database of recent MPXV whole genome sequences described in the previous section for screening of primer binding efficiency was used for subsequent in silico analysis. The sequences were aligned using MAFFT^48^ and the whole target region of the tiled amplicon scheme, excluding the terminal primer binding sites, was extracted from the alignments using Geneious Prime v2025.0.2^50^. The resulting sequences were filtered using Cutadapt^52^ in order to remove sequences with more than 10% ambiguous (N) bases. The filtered sequences were then analyzed using Nextclade (v3.10.0, Mpox virus (All clades) reference dataset)^53^ and the resulting clade assignments were compared to those from GISAID metadata in order to assess the clade differentiation capability and accuracy of the tiled amplicon scheme.

Similar analysis was also performed on each of the 11 individual amplicon regions to assess their capacity to differentiate clades with incomplete target region coverage. Using the MPXV alignments generated previously for whole target region in silico analysis, the primers targeting each amplicon were mapped and each amplicon region, excluding the primer binding sites (coordinates show in Table 1), was extracted using Geneious^50^. The resulting sequences were filtered to remove sequences with any ambiguous bases (N) using Cutadapt^52^. This ambiguous base filtering cut-off was selected because due to the nature of PCR amplification, any amplicons that amplified efficiently should have complete coverage across their entire length. Similar to the whole genome sequence analysis, filtered sequences were analyzed using Nextclade (v3.10.0, Mpox virus (All clades) reference dataset)^53^ and clade assignments were compared to those from GISAID metadata.

### PCR amplification and next generation sequencing

Amplicons were generated using the Q5 Hot Start High-Fidelity DNA Polymerase kit (NEB, Massachusetts, USA; M0493S). For each sample, two PCR reactions were prepared, using either primer pool 1 (containing primers for odd-numbered amplicons) or primer pool 2 (containing primers for even-numbered amplicons) and the products of each primer pool were combined post-amplification. PCR master mixes were prepared using 5 µL of extracted nucleic acid, 12.5 µL of 5X Q5 Reaction Buffer, 1.44 µM of either pool 1 or 2 (3.6µL of a 10 µM pooled primer stock) and nuclease-free water up to a total volume of 25 µL. PCR was carried out at 98 °C for 3 min, followed by 35 cycles of 98 °C for 15 s and 61 °C for 5 min, with a final hold at 4 °C using an Eppendorf Mastercycler Nexus Gradient GSX1 Thermal Cycler (Fisher Scientific, E6332000029). All runs included a MPXV clade IIb positive control sample consisting of diluted AMPLIRUN Monkeypox Virus DNA Control (Vircell Molecular, MBC146-R). Additionally, non-MPXV *Orthopoxvirus* positive control DNA (vaccinia virus Western Reserve (NML collection, initially provided by Dr. David Evans, University of Alberta), was amplified and sequenced to confirm that sequencing results could be differentiated from MPXV.

The PCR products were run on a 4200 Tapestation System (Aglient Technologies, G2991BA) with a D5000 ScreenTape System (Agilent Technologies, 5067–5588, 5067–5589, 5067–5590) for confirmation of successful amplification following the standard manufacturer’s protocol. Quantification of each sample was done with a Qubit dsDNA Quantification Assay Kit (ThermoFisher Scientific, Q32851) on a Qubit Flex Fluorometer (ThermoFisher Scientific, Q33327) and Picogreen dsDNA Reagent (Invitrogen, P7581) using a FilterMax F5 Multi-Mode Microplate Reader (Molecular Devices, F5). Sequencing library preparation was performed on prepared amplicons using the Illumina Nextera XT library preparation kit as per manufacturer’s instructions. DNA libraries were quantified, pooled and sequenced on an Illumina NextSeq 2000 instrument using a P2 600 cycle kit (Illumina). All sequencing runs included a no template control sample, which underwent PCR amplification and sequencing in parallel with other samples but with water instead of a nucleic acid template.

### Sequence assembly and analysis

Quality and primer trimming, read mapping, consensus sequence generation and clade assignment were performed using the nf-core/viralrecon v2.6.0 pipeline^54^ using MPXV isolate M5312_HM12_Rivers (NCBI accession NC_063383.1), trimmed to the target PCR region excluding external primer binding regions, as the reference sequence (reference FASTA used included in supplementary materials). The pipeline was run using the viralrecon amplicon protocol^54^ and a custom primer BED file containing the positions of the primers relative to the reference in order to enable trimming of primer sequences (primer BED file used included as supplementary materials). Clade assignment using Nextclade was enabled using the hMPXV nextclade dataset included in the pipeline at the time of analysis (v2.12.0). Due to the version of Nextclade included in the pipeline not being the most current, assembled consensus sequences were also analyzed using the browser version of Nextclade (v3.10.0, Mpox virus (All clades) reference dataset) to verify clade and sub-clade assignment calls. To further confirm the presence or absence of MPXV, the resulting consensus sequences were queried against the NCBI core nucleotide database using blastn^55^. Additionally, raw sequencing reads were taxonomically classified using Kraken2^56^ and subsequently visualized using Pavian^57^.

### Phylogenetic analysis

The MPXV genome database of extracted genome sequences corresponding to the 4.2 kb tiled amplicon target region used previously for in silico primer specificity testing, was used for phylogenetic analysis. To remove sequences with any ambiguous bases, the database was filtered with Cutadapt^52^. The resulting sequence database was further filtered with CD-HIT^47^ using a sequence identity threshold of 0.9995 in order to remove redundant or highly similar sequences, resulting in a final database of 19 representative MPXV sequences. These sequences were aligned to wastewater and positive control derived consensus sequences generated by in-house sequencing using MAFFT^48^. A phylogenetic tree was generated using IQ-TREE^58^ on the find best model setting with ModelFinder^59^ (Best-fit model selected: K3Pu + F) with 1000 ultrafast bootstraps^60^ and then visualized using iTOL^61^ with a vaccinia Western Reserve virus consensus sequence (NML collection) chosen as the outgroup for rooting. Subsequent phylogenetic analysis was carried out using only amplicon 4 by extracting the target region from the previously generated alignment of 19 representative MPXV sequences (excluding primer binding regions) followed by the process described above for phylogenetic tree generation (Best-fit model selected: HKY + F) and visualization. Sequences covering the 4.2-kb region were generated using purified DNA from MPXV clade Ib (Pathoplexus accession # PP_0014F6Y.1) provided by the PHAC-NML Special Pathogens unit, MPXV clade IIb (AMPLIRUN Monkeypox Virus DNA Control, Vircell, reference # MBC146-R) and vaccinia virus Western Reserve (provided to PHAC-NML by Dr. David Evans, University of Alberta, Edmonton, Canada).

### SNP analysis to assess single amplicon clade and sub-clade differentiation

To investigate the clade and sub-clade classification ability of amplicon 4, the single nucleotide polymorphisms (SNPs) from the nucleotide alignment used for phylogenetic analysis were visualized using snipit^62^. Based on these results, a list of potential clade or subclade specific SNPs within amplicon 4 was generated. The specificity of the SNPs to each clade or subclade was further investigated by looking at the nucleotide composition at the associated positions using the sequence database of recent MPXV sequences downloaded from GISAID and previously used in this study for assay design and in silico analysis.

## Results

### Recovery of MPOX material from wastewater samples

The recovery of MPOX material from wastewater samples was complicated by the low abundance of this pathogen in the tested municipal wastewater samples. Within the study period, a total of 196 clinical MPOX cases were reported nationally in Canada which represents less than 0.0005% of the total population. As described in this study, several wastewater processing methods were attempted for recovery of sufficient MPXV genomic DNA for a subsequent Illumina sequencing reaction and due to limited available volumes of wastewater samples, it was not possible to perform a detailed side-by-side comparison of each processing method beginning with the same initial wastewater sample. However, six methods were evaluated using positive control nucleic acid (AMPLIRUN Monkeypox Virus DNA Control, Vircell Molecular, MBC146-R) which allowed for an evaluation of the recovery of nucleic acid processing but is not representative of intact virus recovery. Four of these methods did not generate reads mapping to MPXV from any of the tested wastewater samples. A schematic describing all trialed methods is shown in the supplementary materials (Figure S1) and the previously described solids method (Method F, Figure S1) that successfully concentrated sufficient MPXV material for a sequencing reaction is indicated with a black box. All of the wastewater-derived sequences presented in this study were generated using this concentration method.

### qPCR sensitivity analysis

The sensitivity of the qPCR assays targeting the ITR (G2R_G^43^ and G2R_NML^30^ and F3L^44^ genes were evaluated using gBlock^®^ Gene Fragments (Integrated DNA Technologies) containing the relevant PCR target, which were quantified by digital PCR. Although the dilutions tested with digital PCR were initially estimated to be approximately 10,000 cp/uL based on manufacturer-provided information, the absolute concentrations were determined to be 8,044 cp/uL, 5,005 cp/uL, and 6,821 cp/uL for the gene fragments containing the PCR targets for G2R_G, G2R_NML, and F3L, respectively. Consequently, all calculations for generating the standard curves and assessing qPCR assay sensitivity were adjusted to reflect the absolute concentrations obtained via digital PCR.

The PCR efficiencies for each of the assays were within the desired range of 90–110%^63^ with values of 99.6%, 97.0%, and 94.1% for the G2R_G, G2R_NML, and F3L assays, respectively. The log-linear linear correlation and PCR efficiencies of the G2R_G, G2R_NML, F3L assays are shown in Figure S2. The qPCR assays had calculated LOQs ranging from 5.35 to 12.6 copies per reaction while LODs ranged from 1.91 to 4.13 copies per reaction for each assay (Figures S3, S4). Results from these three qPCR assays were used as an initial screening strategy to detect the presence of MPXV DNA in wastewater samples. Wastewater samples were selected for subsequent sequencing if they produced a qPCR Ct value ≤ 38 with any of the three assays.

### In Silico tiled sequencing primer scheme specificity

A total of 1,482 MPXV sequences were retained for in silico analysis following ambiguous base filtering. Using the entire 4.2 kb tiled amplicon target region, clade level calls were consistent with the clade listed on GISAID meta-data for all sequences analyzed. At the subclade level, the amplicon region-derived calls were 100% in agreement with GISAID meta-data for all subclade Ib, IIa and IIb sequences. Two subclade Ia samples (of 80 total) were called only to the clade level (i.e. called as clade I only), resulting in a subclade level accuracy of 97.5% (Table 2 and S2).

**Table 2:** In Silico clade and subclade level call percent accuracy for both the whole target region and individual amplicons. Raw numbers used to calculate percentages are also available in supplementary materials (Table S2).

	Clade	Amplicon											
		All	1	2	3	4	5	6	7	8	9	10	11
Clade level accuracy	Ia	100%	0.0%	100%	97.2%	100%	100%	0.0%	1.2%	0.0%	0.0%	0.0%	0.0%
	Ib	100%	16.7%	100%	0.0%	100%	100%	0.0%	0.0%	0.0%	0.0%	0.0%	0.0%
	IIa	100%	50.0%	100%	0.0%	100%	100%	100%	100%	100%	0.0%	0.0%	0.0%
	IIb	100%	0.0%	96.3%	100%	100%	100%	100%	100%	100%	0.0%	0.0%	0.0%
Subclade level accuracy	Ia	97.5%	0.0%	24.4%	97.2%	98.6%	0.0%	0.0%	1.2%	0.0%	0.0%	0.0%	0.0%
	Ib	100%	16.7%	0.0%	0.0%	100%	0.0%	0.0%	0.0%	0.0%	0.0%	0.0%	0.0%
	IIa	100%	50.0%	100%	0.0%	100%	0.0%	100%	0.0%	100%	0.0%	0.0%	0.0%
	IIb	100%	0.0%	96.3%	100%	100%	96.6%	1.7%	100%	100%	0.0%	0.0%	0.0%

A similar in silico analysis was performed on each of the 11 individual amplicons (Table 2). At the clade level, amplicons 4 and 5 correctly identified all sequences analyzed while amplicon 2 correctly identified all clade Ia, Ib and IIa sequences and over 96% of clade IIb sequences analyzed (1,334 of 1,385 total). At the subclade level, amplicon 4 correctly identified all subclade Ib, IIa and IIb sequences and over 98% of subclade Ia sequences analyzed (69 of 70 total). The remaining amplicons not specifically mentioned had varying degrees of accuracy depending on the clade or subclade of the sample as shown in Table 2.

### Wastewater-derived consensus sequence analysis

**Fig. 2:**
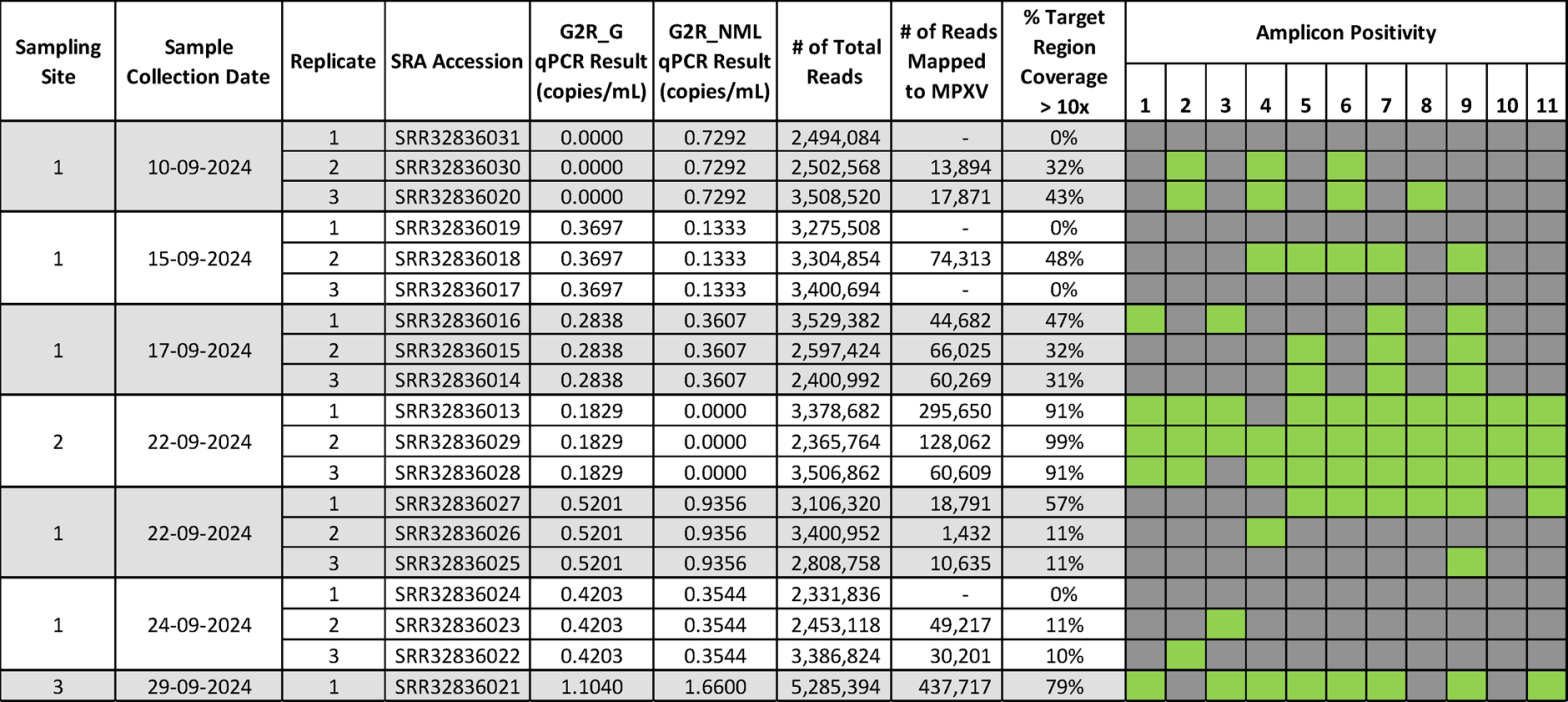
Summary of all MPXV positive samples sequenced in this study.

A total of 23 wastewater samples were processed and sequenced using the method described in this study. Of the 23 total samples, 21 were amplified and sequenced in triplicate, while the remaining two were run in singlicate due to limited sample volume. Seven processed wastewater samples generated a minimum of 1,000 reads mapping to the MPXV reference sequence in at least one of three replicates sequenced (one of these samples was only tested in singlicate). Sample to sample variation included the breadth of target region coverage or total read depth per each amplicon, as shown in Fig. 2. All 15 wastewater-derived MPXV consensus sequences (including individual replicates) were aligned using MAFFT^48^ and found to be identical to each other in overlapping regions. Therefore, a representative high-quality consensus sequence with complete breadth of target region coverage was selected for a more detailed genomic analysis (assembled representative consensus sequence available in supplementary materials). All samples shown in Fig. 2 were sequenced across two sequencing runs, which included both a no template control and a positive control. The first sequencing run, which contained all but the very last sample listed in Fig. 2, the NTC had 0 reads mapping to MPXV following primer trimming. In the second sequence run, the NTC had 4 reads mapping to MPXV outside of primer binding regions. In contrast, all positive wastewater samples shown in Fig. 2 had a minimum of 1,000 reads mapping to MPXV. A consensus sequence generated from clade IIb positive control material is provided in supplementary materials.

**Fig. 3:**
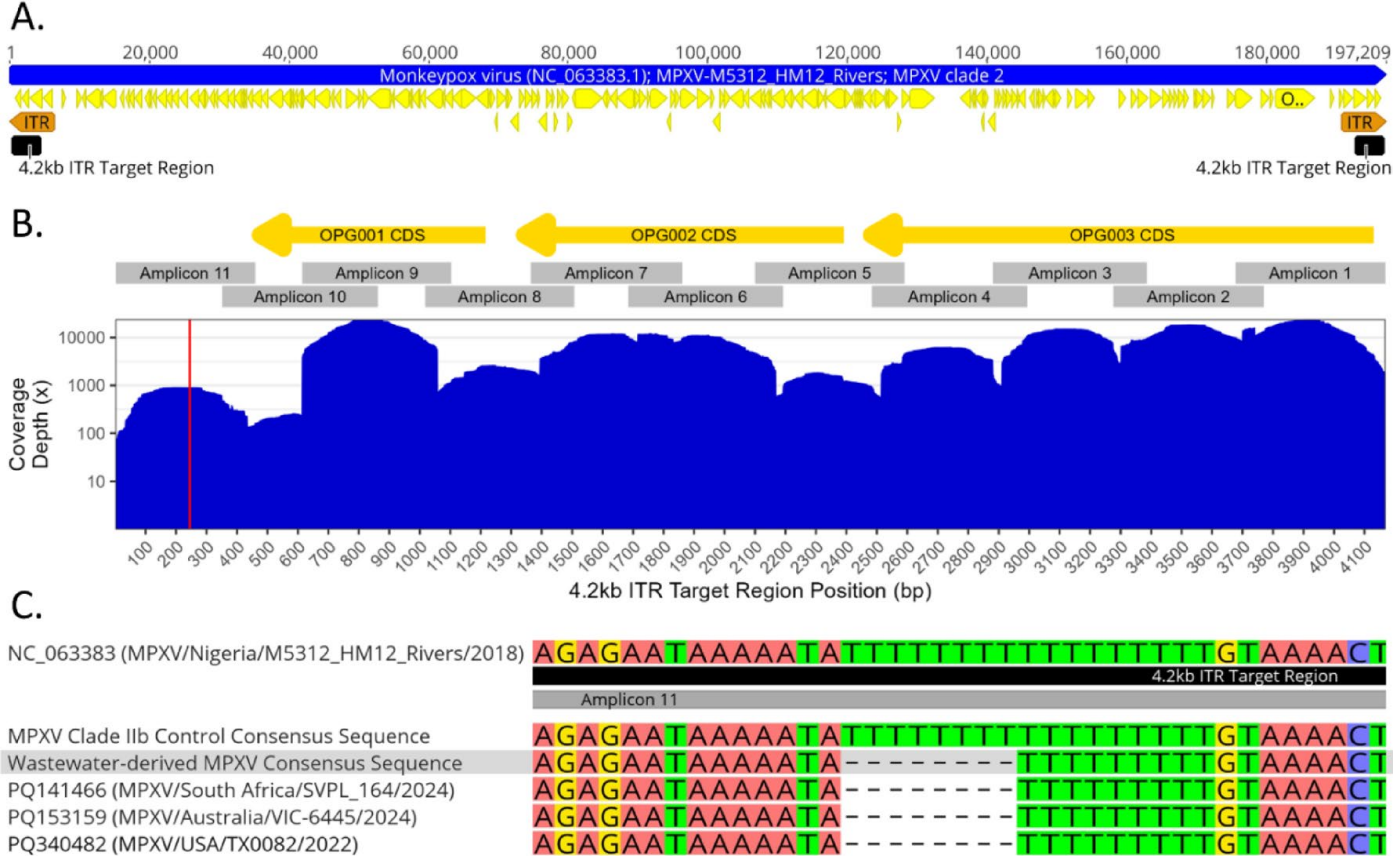
Summary of 4.2 kb ITR tiled amplicon sequencing scheme genomic and coverage information. (**A**) Whole genome schematic of a MPXV clade IIb reference sequence (GenBank accession: NC_063383.1) showing the positions of the ITR regions as well as the PCR target regions. (**B**) Enlarged image of the target region from the 5’ ITR region of the MPXV genome, showing the coding sequences (CDS) in yellow arrows as well as the positions of all 11 amplicons in gray boxes. A coverage plot with the depth of coverage from a representative wastewater sample, which generated a complete sequence across the entire target region is shown (SRR32836029 in Fig. 2). The x-axis represents the genomic position within the target region excluding the terminal primer binding sites and also corresponds to the positions of the CDS and amplicons above the plot. The region highlighted by the red vertical line indicates the location of the homopolymeric region containing the unique deletion relative to MPXV clade IIb control material. (**C**) Further enlarged image showing an alignment of the homopolymeric region with the unique deletion compared to reference sequence NC_063383.1 and MPXV clade IIb control material. The wastewater-derived sequence is highlighted in gray, while other MPXV sequences containing the same deletion are shown with their respective GenBank accession numbers.

A 4,169 bp consensus sequence covering the complete target region was assembled from wastewater-derived sequencing reads. To ensure that the assembled sequence was not the result of contamination from MPXV positive control material included on the same sequencing run, the sequences of the wastewater and positive control derived consensus sequences were aligned. The sequences showed a high degree of similarity, however were distinct based on a homopolymeric stretch within an intergenic region of amplicon 11. While the MPXV-derived positive control had a stretch of 17 consecutive thymidines, the wastewater-derived consensus had a deletion of 8 thymidines (Fig. 3, panel C). This length of this deletion was consistent across all three replicates for this sample, which were PCR amplified, sequenced and analyzed separately. While all three replicates harboured this unique deletion, two of the replicates were missing a single amplicon (Fig. 2) and therefore did not include the entire target region but were otherwise identical in overlapping regions. Based on viralrecon-generated primer-trimmed alignments, the replicate with coverage across the entire target region had a minimum read-depth coverage of 74 and a mean of 8,282 reads (Fig. 3, panel B). A query against the NCBI core nucleotide database (performed January 20, 2025) via blastn^55^ showed that the wastewater-derived MPXV consensus sequence was identical to a number of recent MPXV sequence submissions collected from various locations including the USA, Australia and South Africa which also harbour this 8 nucleotide deletion (Fig. 3, panel C).

### Phylogenetic analysis

**Fig. 4:**
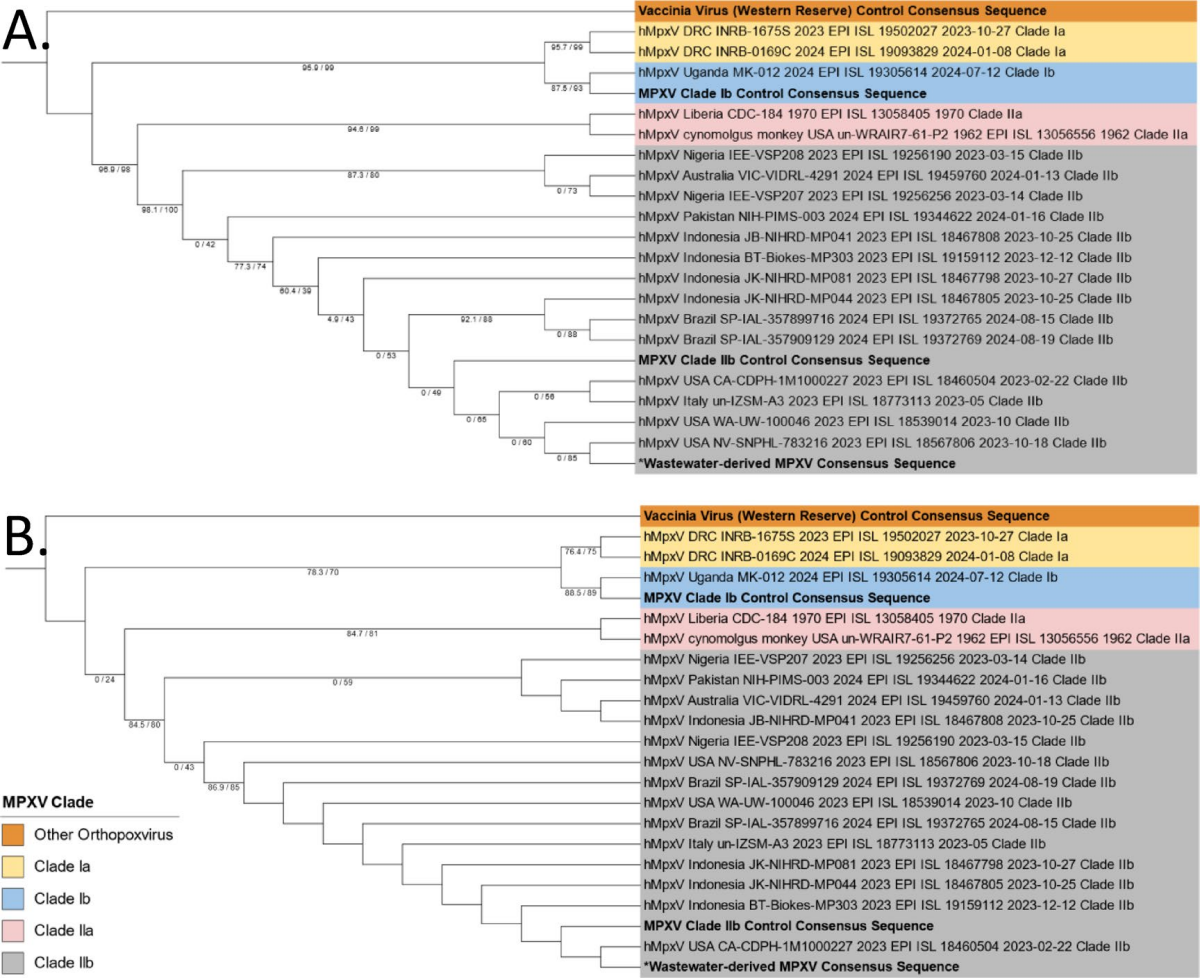
Outgroup rooted phylogenetic trees of the wastewater-derived MPXV consensus sequence and other representative sequences for (**A**) the entire 4.2 kb target region and (**B**) amplicon 4 only. In-house generated sequences from MPXV clade Ib and IIb as well as a vaccinia virus control material are also shown along with a selection of reference sequences from GISAID. SH-aLRT and ultrafast bootstrap (UFBoot) support percentage values, as calculated by IQ-TREE, are shown on each branch in that order (i.e. SH-aLRT/ UFBoot). All in-house generated consensus sequences are bolded with the wastewater-derived sequence indicated with an asterix.

In-house generated consensus sequences of the target region from MPXV clade Ib, MPXV clade IIb and vaccinia virus Western Reserve material as well the wastewater derived MPXV sequence were included in phylogenetic analysis with a subset of MPXV sequences downloaded from GISAID (Fig. 4, Panel A). All samples included in the analysis clustered as expected, with the vaccinia virus and each individual MPXV subclade forming distinct clade-specific branches within the phylogenetic tree. The wastewater-derived consensus sequence clustered with the clade IIb sequences. This result is consistent both with the results of other sequence analysis presented in this study and the expected clade from the sampling site based on reported clinical cases in Canada^64^, which with the exception of a single case reported in November 2024, have been uniquely typed as clade II. Additional phylogenetic analysis was performed with amplicon 4 in order to assess the ability of this amplicon individually to characterize MPXV samples and also to mitigate the effect of repetitive sequences, such as the homopolymeric region contained in amplicon 11. Phylogenetic results for amplicon 4 were in agreement with the genetic relationships observed in phylogenetic analysis of the entire target region with each individual MPXV subclade forming distinct clade-specific branches.

### Clade and sub-clade identification analysis of amplicon 4 SNPs

**Fig. 5:**
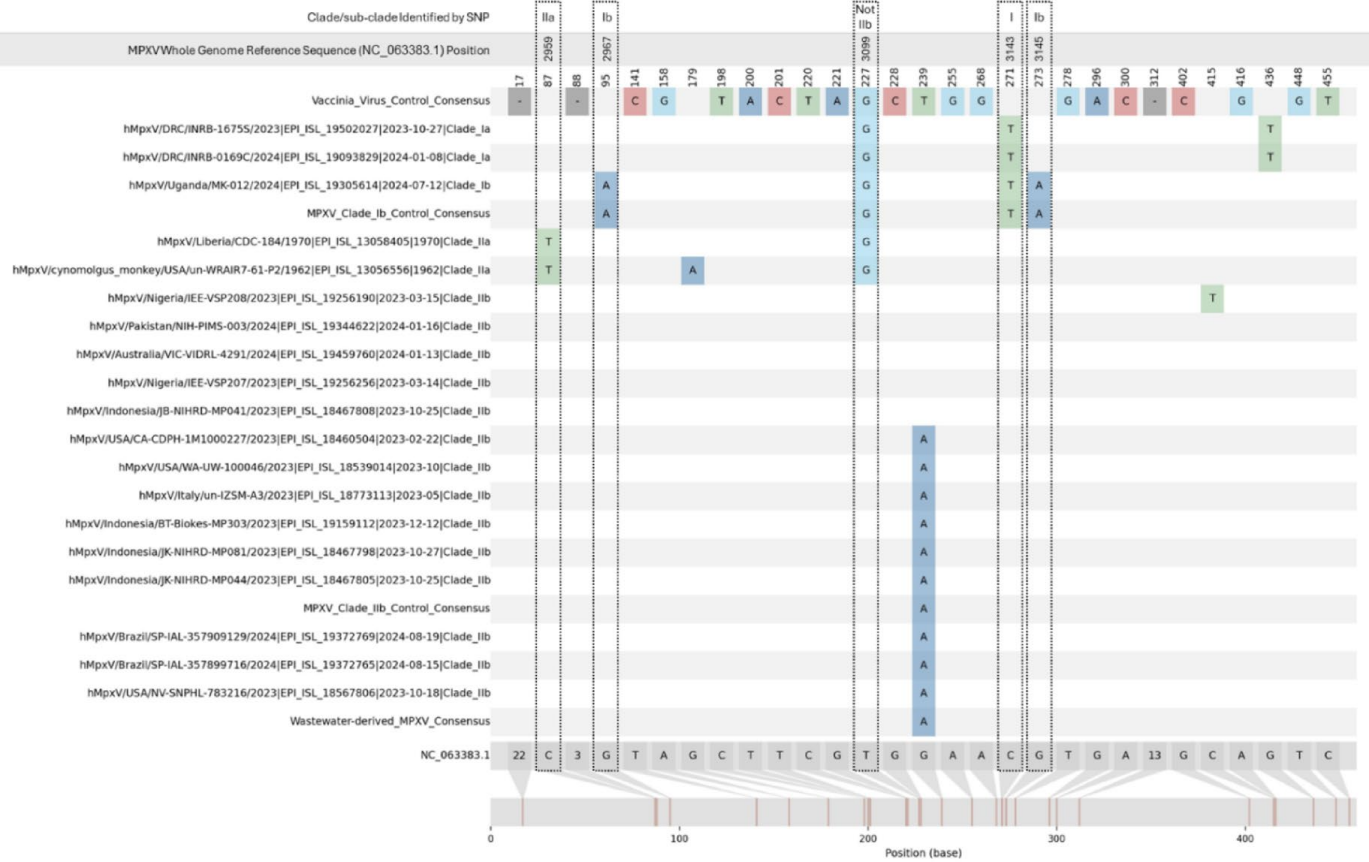
Summary of clade and sub-clade specific single nucleotide polymorphisms (SNPs) in amplicon 4 of representative MPXV sequences. Visualization was carried out using the nucleotide sequence alignment generated for amplicon 4 phylogenetic analysis. All SNPs are relative to the MPXV clade IIb reference sequence (NC_063383.1) which is shown at the bottom on the plot. Within the plot, colored nucleotide bases in non-reference sequences indicate SNPs and dashes indicate deletions relative to the reference sequence. Annotations on the reference sequence indicate the nucleotide base at the position within the reference and numbers indicate the size of the deletion present at that position in at least one of the non-reference sequences. Unlabelled points in the plot on non-reference sequences indicate that the corresponding sequence matches the reference at that position. On the left side of the plot, sequence names are listed with sequences downloaded from GISAD also including accession number, collection date and sub-clade identity. Potential clade or sub-clade specific SNPs are outlined on the plot with a black dotted line. Labelled above the plot, starting from top to bottom, is the clade or sub-clade a SNP is specific to, the position of the SNP relative to the whole genome reference sequence and finally the position of the SNP within the amplicon 4 alignment used for visualization.

Based on analysis of amplicon 4, a total of 5 SNPs were identified that appeared to be clade or subclade specific (Fig. 5). A single SNP was capable of differentiating clade I from clade II (C3143T relative to reference NC_063383.1). The remaining 4 SNPs were subclade specific with 2 associated with Ib (G2967A and G3145A), 1 with IIa (C2959T) and 1 with non-IIb (T3099G). Investigation of these positions in the database of recent MPXV sequences used for assay design and in silico analysis showed the SNPs appear to be specific to the clade or sub-clade designation shown in Fig. 5. C3143T was present in all 121 clade I samples and absent in all 1,392 clade II samples included in the database that had sequence at this position. For the clade Ib specific mutations, both G2967A and G3145A were present in all 13 Ib sequences and absent from the remaining non-Ib sequences (1,501 and 1,499, respectively). C2959T was present in the 2 historical clade IIa sequences included in the database and absent in the remaining 1,513 non-clade IIa sequences. Finally, the non-clade IIb specific T3099G was present in all 134 non-clade IIb sequences and absent in all 1,390 clade IIb sequences. These results show that the SNPs identified within amplicon 4 appear to be specific to their associated clade or subclade in recent MPXV sequences and could potentially be used in combination for MPXV sample characterization.

## Discussion

This study describes a start-to-finish laboratory workflow for the extraction and sequencing of low-abundance MPXV from complex wastewater samples. Previously published qPCR assays were employed as a rapid screen to determine whether MPXV could potentially be sequenced from wastewater samples. This screening approach advantageously reduces the risk of performing costly PCR and sequencing reactions on samples that do not contain MPXV. Coupled with a novel tiled amplicon sequencing approach targeting the genetically variable ITR region, this method was used to generate epidemiologically informative sequences permitting clade and subclade level identification of wastewater-derived MPXV sequences. A 4.2 kb portion of the ITR was selected as the target region for two major reasons; first, this region contains lineage-delineating mutations that allow for MPXV clade and subclade assignment, and second, there are two copies per genome, which effectively doubles the amount of viral starting material. This duplication was important due to the low quantity of MPXV in wastewater, resulting from the significantly reduced clinical incidence of MPXV compared to other viruses currently circulating in the Canadian population, such as SARS-CoV-2, which are also shed into the wastewater. In contrast to whole genome sequencing approaches, only a portion of the genome was targeted due to the low quantity of MPXV in wastewater samples, as well as the high propensity for primer dimer formation when using the large number of primer pairs required to tile across a genome of approximately 197 kb (estimated at a minimum of 500 primer pairs with an amplicon length of 400 bp). Importantly, this assay was developed in response to a lack of recovered MPXV sequences from wastewater samples using published whole genome sequence assays designed for clinical sequencing^65^.

The entire 4.2 kb tiled amplicon target region, composed of 11 separate amplicons, was successfully used to characterize MPXV consensus sequences derived from municipal wastewater to both the clade and subclade level. Based on the results of in silico analysis, all sequences included were correctly called to the clade level. At the subclade level, all clade Ib, IIa and IIb sequences and over 97% of clade Ia sequences were correctly identified. Since wastewater samples typically contain only low concentrations of viral nucleic acid and contaminants which may inhibit amplification and sequencing, the clade and subclade differentiation capability with only partial target region coverage was also assessed. In silico analysis performed separately on each of the 11 amplicons showed that for clade level differentiation, amplicons 4 and 5 were the most accurate and identified all sequences included in the analysis. Amplicon 2 also showed high clade level accuracy, correctly identifying 100% of clade Ia, Ib and IIa sequences and over 96% of clade IIb sequences. For subclade level differentiation amplicon 4 was the most accurate, correctly calling the subclade of all Ib, IIa and IIb sequences and over 98% of clade Ia sequences. Despite the limited clade and subclade differentiation ability of the less specific amplicons, they still may provide valuable genomic information. For example, amplicon 11 contained a deletion in a homopolymeric region of the wastewater-derived consensus sequence which allowed for source attribution and differentiation from MPXV control material.

The differentiation capacity of individual amplicons is important to note in the context of emerging variants, as the ITR regions are prone to recombination which could impact the assay sensitivity. To investigate the impact of mutations in this region, two sequences with large deletions in their ITR regions such as Genbank sequences OP526855, MT903341. were included in the in silico analysis. Although amplicon drop-outs are predicted to occur for each sequence, these deletions are not predicted to cause complete assay failure as other amplicons would still be amplified. This is promising as we have found that MPXV is difficult to recover and sequence from low abundance wastewater samples, and a key advantage of the tiled amplicon scheme targeting the ITR region, is increased template for sequencing due to the duplicate presence of this genomic region. Additionally, a multiple amplicon tiled approach offers the advantage of retaining the ability to generate sequence from other amplicons even when a primer binding site from another amplicon is mutated or disrupted by a deletion. Not unlike other tiled amplicon assays designed and in use for tiled amplicon sequencing of SARS-CoV-2, we anticipate that as new variants achieve dominance amongst circulating strains, that certain tiles of this assay may in response, require modification of the primer sequences. It is important to monitor the evolution of this virus for emergence of new strains impacting the sensitivity of this assay. Subsequent in silico analysis of new clinical sequencing data is recommended to guide this occurrence.

The capability of the tiled amplicon sequencing method to identify and characterize MPXV in practice was demonstrated through the assembly of MPXV consensus sequences from wastewater samples. The presence of MPXV was verified by Kraken2^56^ taxonomic classification of raw sequencing reads as well as Nextclade^53^ analysis and a blastn^55^ query of the MPXV consensus sequence assembled via viralrecon^54^ analysis. Although positive MPXV control material was included on the same run, differences in the length of a homopolymeric stretch between consensus sequences supported the wastewater-derived consensus sequence being from a distinct source. The wastewater-derived MPXV consensus sequence was identified as clade IIb based on results from Nextclade^53^, phylogenetic analysis and blastn^55^ query results. These results are also consistent with clinical data available from the collection area which are solely classified as clade IIb. To date Canada has only reported a single clinical clade Ib case which was from a region not included in this analysis.

In summary, we have described the development, validation and deployment of a new tiled amplicon sequencing approach following the concentration and extraction of MPXV nucleic acid from wastewater samples. While this manuscript was in preparation, the authors became aware of approaches under development for tiled amplicon sequencing of MPXV from wastewater^66,67^. However, to the best of our knowledge, this is the first description of a tiled amplicon sequencing method that enables clade and subclade determination of MPXV from municipal wastewater samples. Although the ability to recover and sequence this virus from wastewater was impacted due to the low abundance in the tested wastewater samples, we have identified the future potential to develop an assay targeting the amplification of a 500-1,000 nt region corresponding to amplicons 4 and 5 for detection and clade identification of mpox from wastewater samples which may increase assay sensitivity. In conclusion, our study contributes to the literature by describing a low-cost, low-complexity protocol for concentrating a large DNA virus from wastewater, followed by tiled amplicon sequencing of an epidemiologically informative genomic region of a low abundance pathogen.

## Supplementary Information

Below is the link to the electronic supplementary material.

Supplementary Material 1

Supplementary Material 2

Supplementary Material 3

Supplementary Material 4

Supplementary Material 5

Supplementary Material 6

Supplementary Material 7

Supplementary Material 8

Supplementary Material 9

Supplementary Material 10

Supplementary Material 11

Supplementary Material 12

## Data Availability

All raw wastewater sequencing data generated for this study using the method described, is available via the NCBI Sequence Read Archive under the BioProject ID PRJNA1241250 available at: https://www.ncbi.nlm.nih.gov/bioproject/PRJNA1241250.
