# Supplementary material for: A targeted tiled amplicon sequencing approach for clade and subclade level differentiation of monkeypox virus from wastewater
Source: Sci Rep. 2025 Aug 11;15:29361. doi: 10.1038/s41598-025-13927-y (PMC12340014; doi:10.1038/s41598-025-13927-y)
Supplement: Supplementary file 2 — Supplementary Material 2 [file 41598_2025_13927_MOESM2_ESM.pdf]

SUPPLEMENTAL TABLE

Data Availability

GISAID Identifier: EPI_SET_250624tz
DOI: https://doi.org/10.55876/gis8.250624tz

All genome sequences and associated metadata in this dataset are published in GISAID's EpiPox database. To view the contributors of each individual sequence with details such as accession number, Virus name, Collection date, Originating Lab and Submitting Lab and the list of Authors, visit 10.55876/gis8.250107tk

Data Snapshot

EPI_SET_250624tz is composed of 1,488 individual genome sequences.
The collection dates range from 1962 to 2024-10-22;
Data were collected in 35 countries and territories.
