# Supplementary material for: A targeted tiled amplicon sequencing approach for clade and subclade level differentiation of monkeypox virus from wastewater
Source: Sci Rep. 2025 Aug 11;15:29361. doi: 10.1038/s41598-025-13927-y (PMC12340014; doi:10.1038/s41598-025-13927-y)
Supplement: Supplementary file 7 — Supplementary Material 7 [file 41598_2025_13927_MOESM7_ESM.docx]

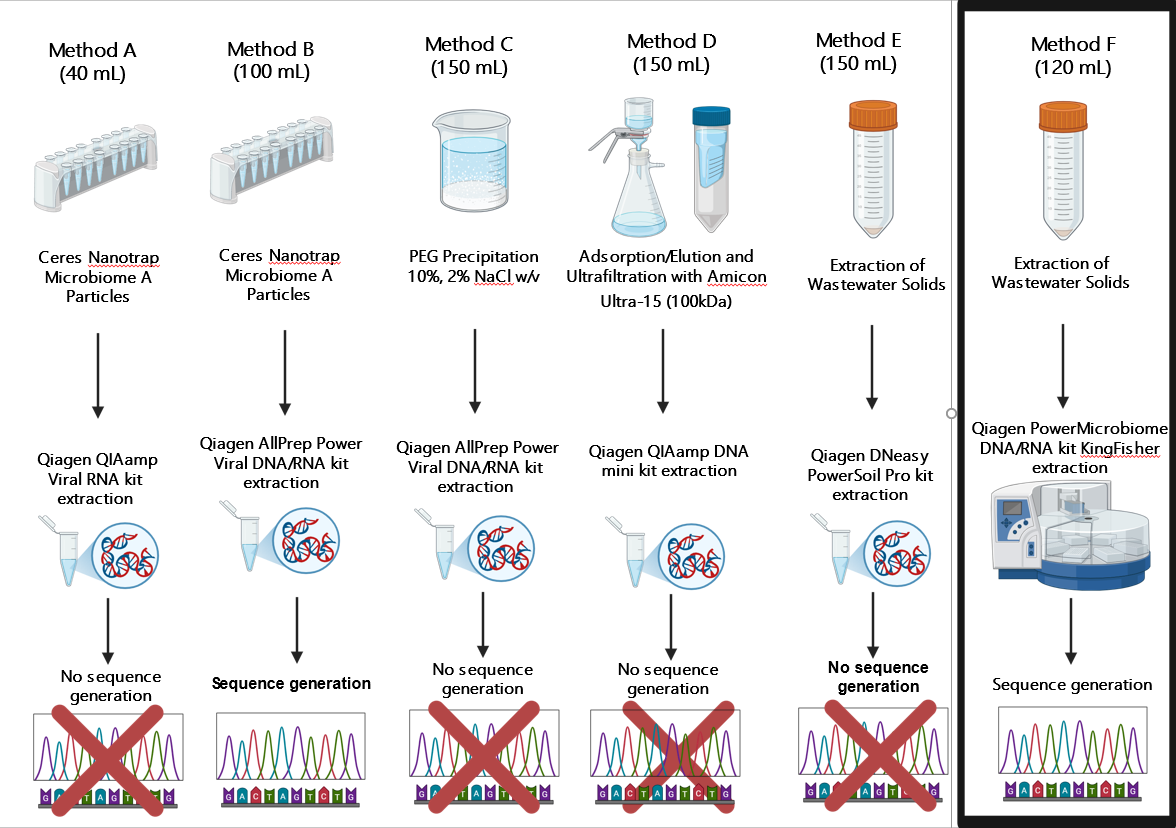


**Figure S1. Wastewater processing methods for the concentration of mpxv from wastewater samples**

**Method A**: Nanotrap® Microbiome A particles (Ceres, Nanosciences) using 40 mL wastewater. This method was used for the affinity capture of mpxv from wastewater samples followed by nucleic acid extraction with the Qiagen QIAamp Viral RNA kit (Cat. No. 52904) and was performed following Ceres Nanosciences protocol, APP-096, Revision 0, December 2022 with the following modifications. Briefly, 600 µL of Ceres Nanotrap microbiome A particles were added to 40 mL of collected wastewater influent, incubated at room temperature for 20 minutes, then separated from the liquid phase by magnetic separation for 10 minutes. Following the separation of the particles from the wastewater, the liquid fraction was discarded, leaving the particles attached to the walls of the collection tube. The mpxv material was next lysed from the particles using lysis buffer (560 µL AVL Buffer and 140 µL 1xPBS), placed onto a magnetic rack for 5 minutes to separate the beads from the sample. The supernatant was carefully pipetted into a new collection tube and nucleic acid was extracted using the Qiagen QIAamp Viral RNA kit (Cat. No. 52904) following manufacturer’s instructions with a final elution into 50 µL of Qiagen EB buffer.

**Method B:** Wastewater was processing similar to Method A with the following modifications. The starting volume of wastewater influent was increased to 100 mL and divided into two aliquots. 600 µL of Nanotrap® Microbiome A particles (Ceres, Nanosciences) was added to each 50 ml aliquot and processed following manufacturer’s instructions. At the final lysis step, 600 µL of lysis buffer was added to the first aliquot, briefly vortexed to lyse the mpxv from the particles, then transferred to the second aliquot and vortexed prior to separation on a magnetic block for 5 minutes. The supernatant was carefully pipetted into a new collection tube and total RNA was extracted using the Qiagen AllPrep Power Viral DNA/RNA kit (Cat. No. 28000) instead of the QIAamp Viral RNA kit (Cat. No. 52904). Sequencing via Illumina NextSeq 2000 generated reads mapping to mpxv however, these were not further included in this study due to the limited number of samples available for processing.

**Method C**: Polyethylene glycol (PEG 8000) precipitation.

This method is frequently described in the literature for the concentration of viruses from various water matrices and samples were processed using a previously described method for recovery of mpxv from wastewater samples with the following modifications. Briefly, 150 mL of wastewater was treated with 10 % PEG-8000 and 2 % w/v NaCl as previously described but the precipitation step was increased to an overnight step at 4 °C and then centrifuged for 100 minutes at 12,000 x g to obtain a more solid pellet. The supernatant was then discarded and the pellet resuspended in 600µL of Qiagen buffer PM1 prior to extraction of nucleic acids using the Qiagen AllPrep Power Viral DNA/RNA kit (Cat. No. 28000) with a final elution into 100 µL of Qiagen buffer EB.

**Method D**: Adsorption/Elution and Ultrafiltration.

Initially, 150 mL of wastewater influent was filtered through a 0.2 micron filter (VWR, Cat. No. EA28147-979). The filtrate was concentrated by ultrafiltration using an Amicon™ Ultra-15 100 kDA centrifugal filter unit (Millipore, Cat. No. UFC-9100) at 4,000 x g for 20 minutes. Adsorption-elution (AE) of 150 mL of wastewater influent was performed following a published method with the following modifications. Wastewater processing was done using an increased volume of 150 mL instead of 50 mL and the ultrafiltration step was performed with an Amicon™ 100 kDa ultra centrifugal filter instead of a 30 –kDa ultra centrifugal filter to concentrate the wastewater to 200 µL. Nucleic acid extraction was performed using the Qiagen QIAamp DNA Mini Kit (Cat. No. 51304) with a final elution into 50 µL of Qiagen buffer EB.

**Method E:** Processing and extraction of wastewater solids.

Due to the lack of concentrated sample and generation of mpxv sequencing reads using the above methods, we attempted to increase the recovery of mpxv from wastewater by capture and extraction of total wastewater solids. Briefly, 120 mL of wastewater was centrifuged for 30 minutes at 12, 000 x g instead of at 4,198 x g for 20 minutes to generate a more solid pellet. The supernatant was removed and the pellet was lysed by addition of 800 µL of CD1 buffer at room temperature followed by nucleic acid extraction using the Qiagen DNeasy PowerSoil Pro kit (Cat. No. 47014) with a final elution into 100 µL of Qiagen buffer EB.

**Method F:** Modified Processing and extraction of wastewater solids.

Finally, extraction of total wastewater solids from 120 mL of wastewater was performed as described in Method E with the following modification of extracting nucleic acid using the Qiagen MagAttract PowerMicrobiome DNA/RNA KF Extraction kit (Cat. No. 27600-4-KF) with final elution into 100 µl of buffer EB. To improve the sequencing yield, a clean-up step was added using a 1:1 ratio of AMPure XP beads for DNA cleanup (Beckman Coulter, Cat No. A63881) prior to the sequencing reaction.

Once processed, RT-qPCR targeting the G2R and F3L genes was performed as described in the manuscript and samples with Ct of ≤ 38 were selected for further processing. Following tiled amplicon enrichment of the 4.2 kb region of the ITR following the procedure described in this study, DNA libraries were generated using the Illumina Nextera XT DNA library preparation kit with 0.2 ng of input DNA and paired end sequencing was performed on an Illumina NextSeq 2000. Although a limited set of mpxv reads were generated using Method B, due to the limited volume of available wastewater for testing, and for method consistency, only sequences generated using Method F are included in this study.
