# Supplementary material for: A targeted tiled amplicon sequencing approach for clade and subclade level differentiation of monkeypox virus from wastewater
Source: Sci Rep. 2025 Aug 11;15:29361. doi: 10.1038/s41598-025-13927-y (PMC12340014; doi:10.1038/s41598-025-13927-y)
Supplement: Supplementary file 8 — Supplementary Material 8 [file 41598_2025_13927_MOESM8_ESM.docx]

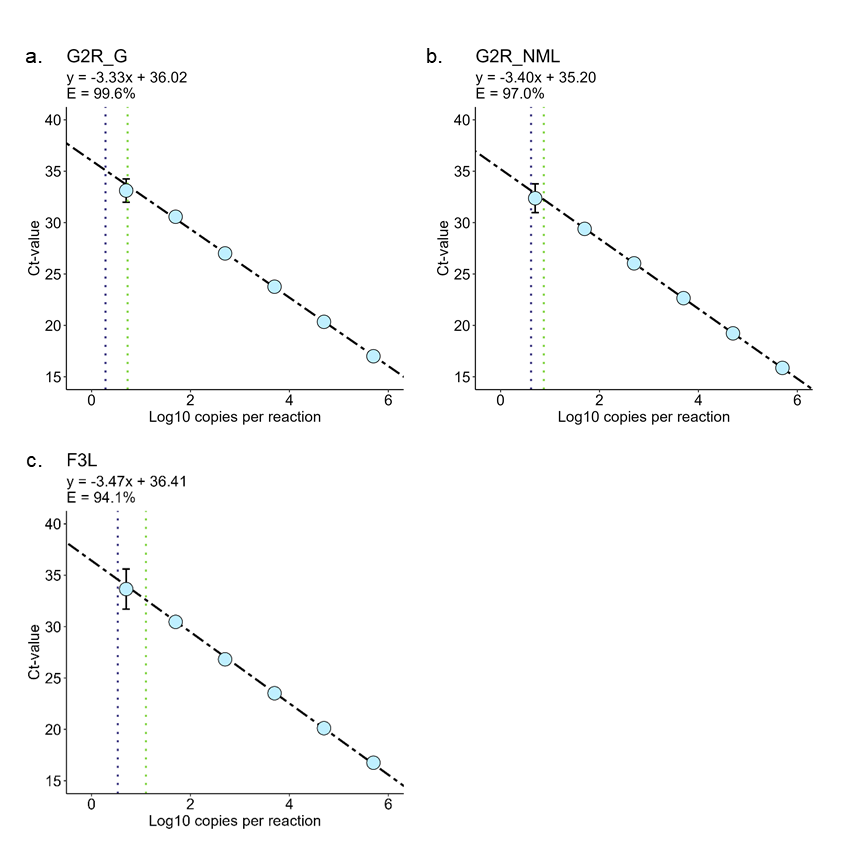


**Figure S2.** Calibration curves generated with a logistic model of Ct-values for a six-fold serial dilution of known concentration of DNA standard. (a) G2R_G, (B) G2R_NML, and (c) F3L. Error bars represent the standard deviation of qPCR replicates. Dotted purple line indicates assay limit of detection. Dotted green line indicates assay limit of quantification. E; PCR efficiency.
