# Supplementary material for: A targeted tiled amplicon sequencing approach for clade and subclade level differentiation of monkeypox virus from wastewater
Source: Sci Rep. 2025 Aug 11;15:29361. doi: 10.1038/s41598-025-13927-y (PMC12340014; doi:10.1038/s41598-025-13927-y)
Supplement: Supplementary file 9 — Supplementary Material 9 [file 41598_2025_13927_MOESM9_ESM.docx]

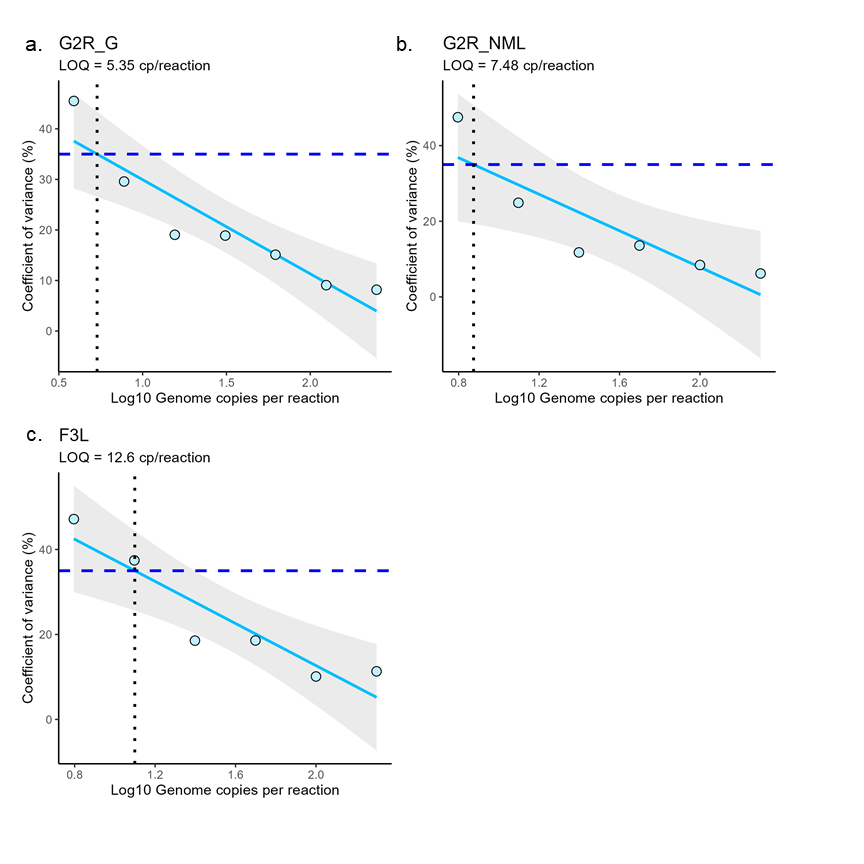


**Figure S3.** Limit of quantification (LOQ) of the G2R_G (a), G2R_NML (b), and F3L (c) qPCR assays. Coefficient of variance calculated from a 10-fold serial dilution fitted to a linear regression model for estimation of the LOQ (solid blue line). Grey band indicates the 95% confidence interval of the regression fit. Black dotted line indicates when the coefficient of variance equals 35% (dashed blue line).
