# Supplementary material for: A targeted tiled amplicon sequencing approach for clade and subclade level differentiation of monkeypox virus from wastewater
Source: Sci Rep. 2025 Aug 11;15:29361. doi: 10.1038/s41598-025-13927-y (PMC12340014; doi:10.1038/s41598-025-13927-y)
Supplement: Supplementary file 10 — Supplementary Material 10 [file 41598_2025_13927_MOESM10_ESM.docx]

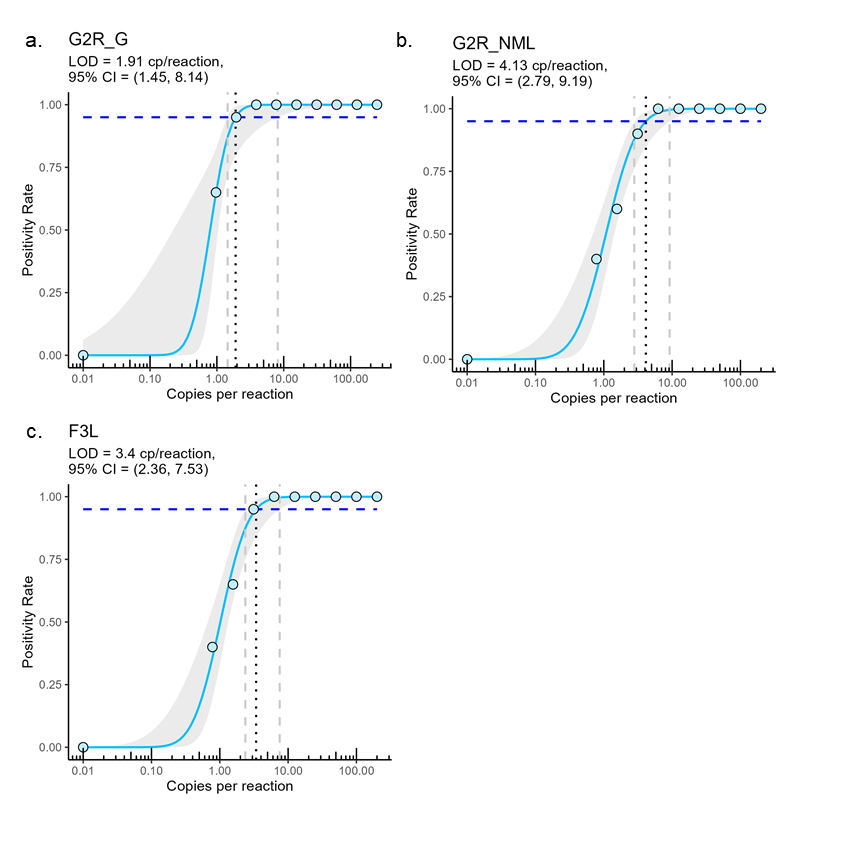


**Figure S4.** Limit of detection (LOD) of the G2R_G (a), G2R_NML (b), and F3L (c) qPCR assays. LOD estimated by fitting positivity rates of a 10-fold serial dilution to a Probit regression (solid blue line). Grey band indicates 95% confidence interval for regression fit. Grey dashed lines indicate the confidence interval (CI) for the LOD. Dashed blue line indicates a positivity rate of 95%.
